# Investigating differential miRNA expression profiling using serum and urine specimens for detecting potential biomarkers for early prostate cancer diagnosis

**DOI:** 10.3906/sag-2010-183

**Published:** 2021-08-30

**Authors:** Sevde HASANOĞLU, Beyza GÖNCÜ, Emrah YÜCESAN, Sezen ATASOY, Yunus KAYALI, Nur ÖZTEN KANDAŞ

**Affiliations:** 1 Experimental Research Center, Bezmialem Vakıf University, İstanbul Turkey; 2 Department of Medical Biology, Faculty of Medicine, Bezmialem Vakıf University, İstanbul Turkey; 3 Department of Biochemistry, Faculty of Pharmacy, Bezmialem Vakıf University, İstanbul Turkey; 4 Department of Urology, Faculty of Medicine, Bezmialem Vakıf University, İstanbul Turkey; 5 Department of Pharmaceutical Toxicology, Faculty of Pharmacy, Bezmialem Vakıf University, İstanbul Turkey

**Keywords:** Prostate cancer, biomarker, microarray, miRNA profiling

## Abstract

**Background/aim:**

MicroRNAs (miRNAs) are known up-to-date candidate biomarkers for several diseases. In addition, obtaining miRNA from different body fluids such as serum, plasma, saliva, and urine is relatively easy to handle. Herein we aimed to detect miRNAs as biomarkers for early stage prostate cancer (PC). For this purpose, we used urine and serum samples to detect any significant differences in miRNA profiles between patients and healthy controls.

**Materials and methods:**

Total ribonucleic acid (RNA) in urine and serum samples were isolated from eight untreated PC patients, thirty healthy individuals were screened for miRNA profile, and candidate miRNAs were validated. Whole urinary and serum miRNA profile was analyzed using Affymetrix GeneChip miRNA 4.0 Arrays. Candidate miRNAs were investigated by stem-loop reverse transcription-polymerase chain reaction.

**Results:**

When we analyzed the urinary samples of PC patients, 49 miRNAs were detected to be upregulated and 14 miRNAs were found to be downregulated when compared with healthy controls. According to the serum samples, 19 miRNAs were found to be upregulated, and 21 miRNAs were found to be downregulated when compared with healthy individuals as well. Interestingly, we detected only four overlapping miRNAs (*MIR320A*, *MIR4535*, *MIR4706*, *MIR6750*) that commonly increase or decrease in both serum and urine samples. Among them, *MIR320A* was found to be downregulated, and *MIR4535*, *MIR4706*, and *MIR6750* were found to be upregulated for urine samples. However, only *MIR6750* was upregulated and the other three miRNAs were downregulated for serum samples.

**Conclusion:**

Notably, the expression profile of *MIR320A* was significantly altered in urine specimens of prostate cancer patients. We considered that *MIR320A* has been evaluated as a valuable biomarker that can be used in the early diagnosis of PC.

## 1. Introduction

Prostate cancer (PC) is the second common cancer in developed countries, and also the most frequent cause of cancer-related mortality in men after lung cancer [1]. The incidence and prevalence of PC varies between different geographical regions, e.g., the most common in North America and less common in South Asia [2]. 

The main diagnostic criteria in PC are digital rectal examination (DRE), serum prostate-specific antigen (PSA) level, and transrectal ultrasound-guided biopsy, respectively. The PSA is exclusively expressed in prostate tissue, yet it is not specific for diagnosis [3]. Increased levels may be associated with numerous clinical conditions such as benign prostate hyperplasia (BPH), prostatitis, other infections, trauma, and urinary retention. [4,5]. Currently, the measurement of PSA is the most common PC indicator [6,7]. However, PC was detected in only 25% of individuals who underwent prostate biopsy based on elevated PSA level (>4.0 ng/mL) [4,5]. In addition, the PSA test has some limitations [4] causing a low positive predictive value (approximately 25%–40%) [4,8]. The role of PSA in prostate cancer has become increasingly controversial [9]. False-negative results and low specificity have limited the application of the clinical use as a diagnostic parameter. 

miRNAs are uncoded RNAs that transcribe gene expression with the average length from 19 to 25 nucleotides [10,11]. Each miRNA binds to the 3’ untranslated region (UTR) of its target mRNA [12], and inhibits the gene expression of multiple targets by binding to more than one region of mRNA’s [13]. 

Recent studies have shown that miRNAs play important roles in many critical biological processes, including development, proliferation, differentiation, apoptosis, tumor formation, signal transduction, organ development, and hematopoietic lineage differentiation [14]. In the literature, there are several studies evaluating different miRNAs as biomarker for early diagnosis [15–18]. Although PSA levels are principally used for diagnosis in PC, despite some controversial cases, additional biomarkers are required to make a definitive diagnosis. For this purpose, the present study demonstrated PC-related miRNAs such as *MIR141*, *MIR21*, *MIR200B*, *MIR221*, and *MIR375*. Particularly, *MIR375*, identified as a urine-associated miRNA, is directly related to stage two PC [19]. Importantly, a recent metaanalysis demonstrated 10 upregulated and 14 downregulated miRNAs that may discriminate PC from BPH/normal control tissues [20]. 

miRNAs have been suggested as a novel biomarker in cancer due to their presence in different body fluids such as serum, plasma, saliva, and urine [21]. They may also be used in the early diagnosis of PC [22]. As urine is one of the most easily accessible biofluids in primary care clinics, a urine-based new biomarker test may help to clarify PC diagnosis. Studies show that miRNAs are a more sensitive marker than PSA level for PC [19]. Furthermore, serum and urinary miRNA expressions, together, may define the diagnosis criteria for PC patients. 

Understanding which miRNAs are present in urine and serum samples when comparing controls and patients may offer a novel insight into the early diagnosis of PC. The aim of this study is to determine a possible biomarker of miRNAs in serum and urine samples of PC patients using microarray expression analysis. Selected candidate miRNAs were investigated and validated in detail for each sample by stem-loop qRT-PCR. 

## 2. Materials and methods

### 2.1. Ethical approval

This study was conducted after receiving approval from the Local Human Ethics Committee (approval number: 16926-22/30) from Bezmialem Vakıf University. All protocols conformed to the ethical guidelines of the Helsinki Declaration and written informed consent was obtained from all subjects.

### 2.2. Donor recruitment

Eight patients with newly diagnosed untreated prostate cancer (mean age, 62 years; range, 49–72 years) who were referred from the Department of Urology were enrolled in this study. The clinical findings of the patients are listed in Table 1. Thirty healthy individuals (mean age, 35 years; range, 20–73 years) who did not have either suspected cancer or any metabolic diseases were used as a control group. Urine samples were collected and used immediately to maintain freshness for further experiments. Blood samples were collected using EDTA-coated tubes (Vacuette, Grenier Bio-One, Austria). Serum samples were separated by centrifugation at 1100 g for 10 min and stored at –80 °C until use. 

**Table 1:** Gleason grading system and PSA levels for patients who have untreated prostate cancer (Gleason score; >7: high grade, 7: intermediate grade, <7: low grade). PSA: prostate-specific antigen.

Patient	Age	Serum PSA level (ng/mL)	Gleason score
#1	52	40	6 (3+3)
#2	49	6.39	6 (3+3)
#3	60	36	9 (5+4)
#4	67	7.6	7 (4+3)
#5	72	3.3	6 (3+3)
#6	65	22.3	7 (3+4)
#7	70	15.11	9 (4+5)
#8	61	30	7 (4+3)

For total RNA isolation from urine samples, freshly collected 50 mL of urine samples were centrifuged at 1210 × *g* for 20 min. The pellet was dissolved in 5 mL of 1X PBS (Phosphate Buffer Solution-Thermo Fisher Scientific, MA, USA) (pH 7.4) and continued through lysis phase. Total RNA isolation was performed according to the manufacturer’s protocol using the commercial kit (Urine Total RNA Purification Maxi Kit, Norgen, Ontario, Canada). 

### 2.3. Total RNA isolation from serum samples

Serum samples were incubated on ice for a complete thaw process. Immediately, triazole reagent (1:1 ratio) (Thermo Fisher Scientific, MA, USA) and nuclease-free glycogen (1 μg/mL) (Thermo Fisher Scientific, MA, USA) were added, respectively. They were then incubated at room temperature for 10 min. Afterward, 200 μL of chloroform (Merck Millipore, Darmstadt, Germany) was added and the samples were incubated for 15 min. Next, they were centrifuged at 12,000 × *g* for 15 min at 4 °C. Upper aqueous phase was transferred, and 1.2 mL of isopropanol (Merck Millipore, Darmstadt, Germany) was added to the samples. After that, the samples were vortexed and then incubated for 10 min at room temperature. They were centrifuged at 12,000 × *g* for 8 min. The supernatants were aspirated carefully. One milliliter of 75% ethanol was added and the samples werecentrifuged at 7500 × *g* for 5 min at room temperature. The supernatant was carefully removed, and the tubes were dried out at room temperature. Each pellet was resuspended with 20 μL of nuclease-free water (AppliChem, Gatersleben, Germany) and stored at –80 °C for further evaluation. 

### 2.4. microRNA expression array

Stored RNA samples were incubated on ice for a complete thaw process. The quantitative analysis of RNA was conducted by measuring optical density at 260 nm and 280 nm using a Multiskan GO (Thermo Fisher Scientific MA, USA) for microarray expression analysis. Between 100 and 300 ng of total RNA samples from the urine and serum control and patient groups were pooled. GeneChip® miRNA 4.0 Array-Affymetrix (Affymetrix, Santa Clara, California, US, catalog number 902411) microarray analysis was performed to identify miRNAs that were significantly differentially expressed. All microarray experiments were performed at Ayka Laboratories (Ayka Medikal, Ankara, Turkey). Identification of differentially expressed miRNAs at each stage was achieved by using linear regression and hierarchical clustering analysis with the Transcriptome Analysis Console software from Affymetrix. The filter settings were as follows: “Transcription Cluster ID”: contains hsa-miR does not contain mmu-miR, “Fold change”: +5 and −5, “False discovered rate (FDR)”: >2.0 or <0.5 “Adjusted P-value threshold”: 0.05.

### 2.5. Stem-loop RT-PCR

A total of 76 samples’ (eight patients and 30 control samples of urine and serum) reverse transcription of 500 ng total RNA was performed using miScript II RT Kit (Qiagen, Hilden, Germany) according to the manufacturer’s protocol. The obtained cDNAs were stored at –20 °C for further evaluation. Real-time PCR for miRNAs was performed on a CFX-connect Real-Time PCR Instrument (Biorad, CA, USA) using miScript SYBR Green PCR Kit and miScript Primer Assay (cat no: MS00014707 and MS00046543) both from Qiagen (Hilden, Germany) according to the manufacturer’s instructions. As an endogenous reference control, RNU6-2 was used [23]. 

The primers were as follows: *hsa-miR-6750-5p*: 5’CAGGGAACAGCUGGGUGAGCUGCU, *hsa-miR-320a*: 5’AAAAGCUGGGUUGAGAGGGCGA. Relative miRNA expression levels were calculated using the 2^-ΔΔ ^CT method. 

### 2.6. Statistical analysis

The false discovery rate (FDR) was set to <0.05 and the minimum fold change (FC) was set to >2.0 or <0.5 for microarray analysis. For the RT-PCR data, statistical analysis was performed using Graph Pad (Prism 7.0, GraphPad Software, Inc., CA, USA). The data were compared using the Mann–Whitney U test and Sidak’s multiple comparisons test. A p-value of <0.05 was considered to be statistically significant. 

## 3. Results

The yield of total RNA from serum and urine specimens was determined, and it showed significant differences among samples of the patients and the healthy control groups. The overall medians of urine and serum samples were 46.5 ng/µL and 483.9 ng/µL, respectively. 

To identify miRNAs differentially upregulated and/or downregulated in PC patients, miRNA microarrays were used to determine the total expression profile. Patients’ and healthy controls’ urine and serum samples were pooled into four independent groups (group 1: patients with serum sample, group 2: patients with urine sample, group 3: healthy controls with serum sample, group 4: healthy controls with urine sample) and were profiled on Affymetrix GeneChip miRNA 4.0 Arrays. Affymetrix GeneChip miRNA 4.0 Array covers of all the 2.578 mature human miRNAs available in miRBase version 20 (June 24, 2013) were used to profile miRNA expressions [24]. 

**Figure 1:**
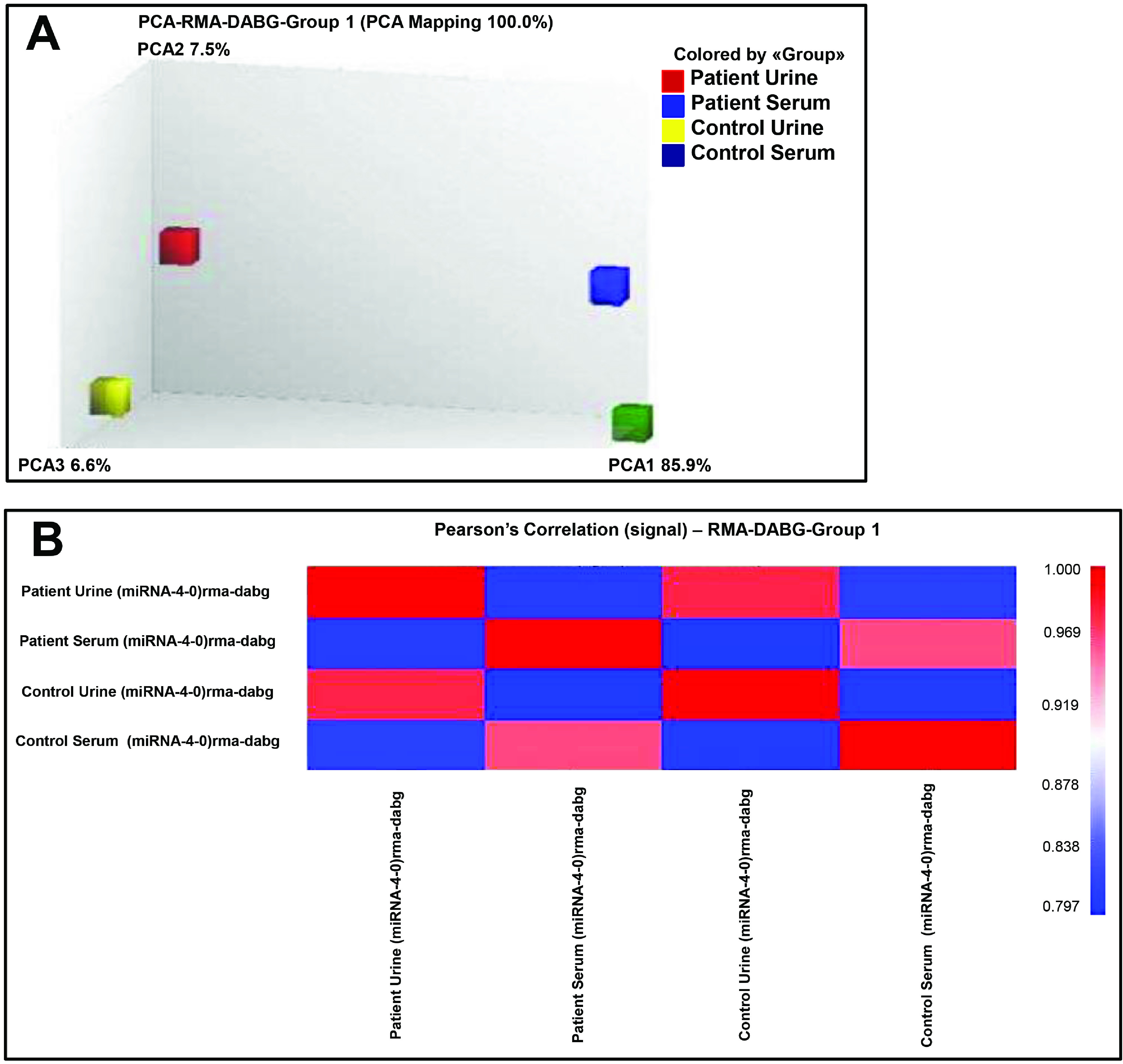
A) Loading plot showing the components from Principal Component Analysis (PCA) of all probe sets from Affymetrix GeneChip miRNA Arrays 4.0 hybridized. The observed four groups are shown (red cube: patient urine samples, blue cube: patient serum, yellow cube: control urine and green cube: control serum). B) Pearson’s correlation of the signal obtained from Affymetrix miRNA Arrays 4.0 GeneChip® hybridized.

**Figure 2:**
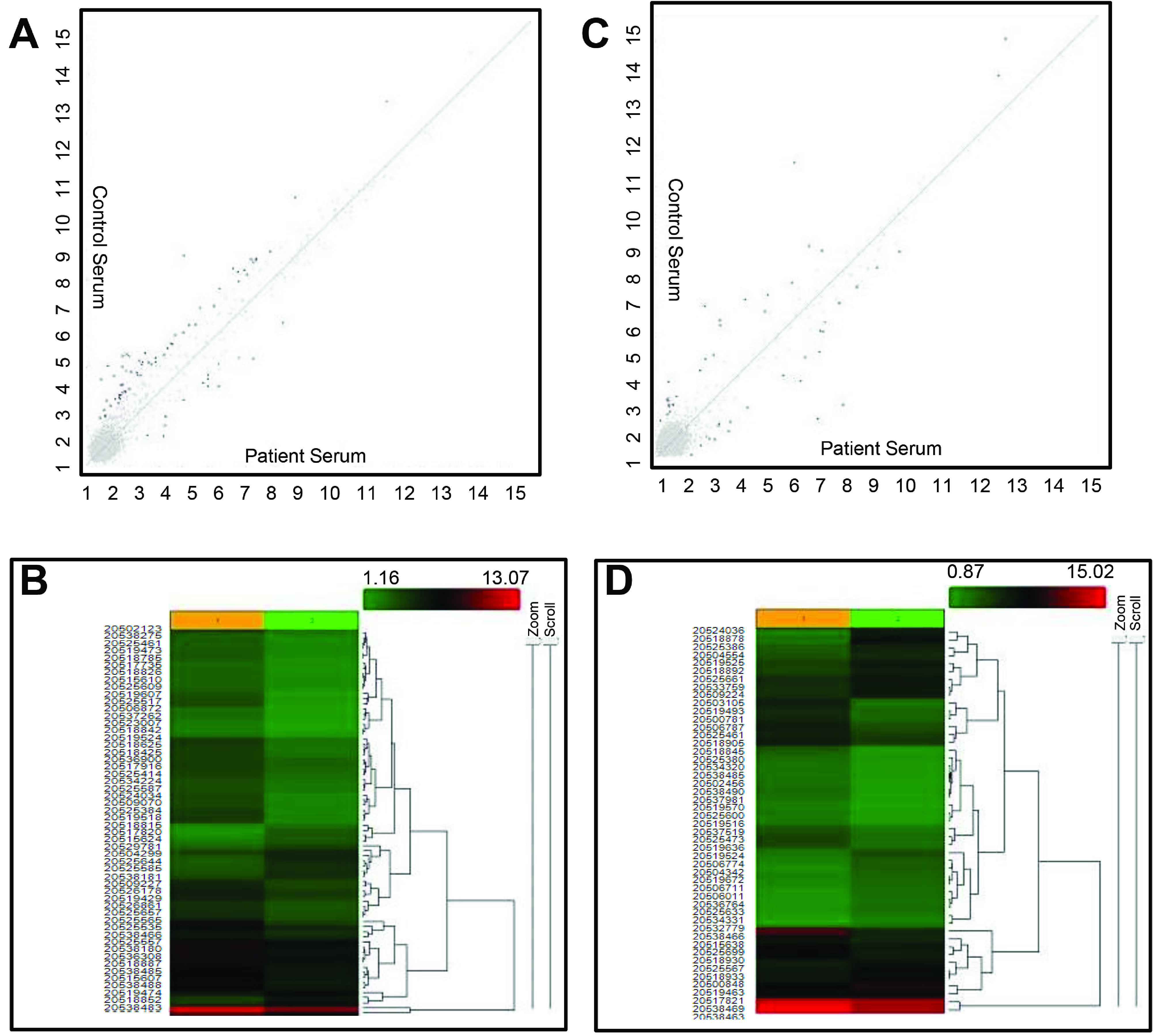
A) Scatter plot from GeneChip miRNA Arrays 4.0 analysis. The expression profiles of urine samples comparing patient and control groups were plotted. Significant spots in red and green represents the number of miRNAs in urine samples which are upregulated and downregulated, respectively (Y axis shows expression levels in control urine samples, X axis shows expression levels in patient urine samples. B) Scatter plot from GeneChip miRNA Arrays 4.0 analysis. The expression profiles of serum samples comparing patient and control groups were plotted. Significant spots in red and green represents the number of miRNAs in serum samples which are upregulated and downregulated, respectively (Y axis shows expression levels in control serum samples, X axis shows expression levels in patient serum samples. C) Heat map from GeneChip miRNA Arrays 4.0 analysis of urine samples. The clustering of samples was mainly divided into two major clusters; one was the control samples and the other was patient samples. Adjusted p-value threshold: 0.05. Red represents the upregulated miRNA while green represents the downregulated miRNA respectively. D) Heat map from GeneChip miRNA Arrays 4.0 analysis of serum samples. The clustering of samples was mainly divided into two major clusters; one was the control samples and the other was patient samples. Adjusted p-value threshold: 0.05. Red represents the upregulated miRNA while green represents the downregulated miRNA, respectively.

The miRNA QC tool was used to assess the quality of the array data and the principal component analysis (PCA) mapping shown in Figure 1A. Afterwards, the data were normalized by global normalization with robust multichip average (RMA) and detection above background (DABG) using the Expression Console software (Affymetrix, Santa Clara, CA, USA) (Figure 1B). The differentially expressed miRNAs between patient and control groups of urine and serum samples are shown in the form of a scatter plot in Figures 2A and 2B. In order to better characterize the differential miRNAs to distinguish between patient and healthy control samples, the miRNAs were further used to heat map clustering analysis and urine and serum sample clusters shown in Figures 2C and 2D, respectively. The mature human miRNAs represented on the microarray showed that 103 (3.99%) miRNAs were expressed totally in all groups (patient and healthy control groups from urine and serum samples). There were 49 (1.9%) and 14 (0.54%) up- and downregulated urine miRNAs, respectively, when the patient and healthy control groups were compared (Table 2). There were 19 (0.73 21 (0.81%) up- and downregulated serum miRNAs (Table 2), when the patient and healthy control groups were compared. 

**Table 2:** Transcript ID and fold change of miRNAs in urine and serum specimens of patients and healthy groups. Overlapping miRNAs both for serum and urine specimens are marked as bold. –: downregulated, +: upregulated.

miR ID	Accession #	Specimens	Fold Change
MIR4532	MIMAT0019071	Urine/Serum	+3.99/–2.05
MIR6750	MIMAT0027400	Urine/Serum	+2.23/+2.37
MIR4706	MIMAT0019806	Urine/Serum	+2.66/–2.72
MIR320A	MIMAT0000510	Urine/Serum	–4.2/–2.05
MIR744	MIMAT0004945	Urine	+6.21
MIR6726	MIMAT0027353	Urine	+5.49
MIR4485	MIMAT0019019	Urine	+5.34
MIR6813	MIMAT0027526	Urine	+5.24
MIR3180–4	MIMAT0018178	Urine	+4.33
MIR3197	MIMAT0015082	Urine	+4.24
MIR4532	MIMAT0019071	Urine	+3.99
MIR4674	MI0017305	Urine	+3.57
MIR4449	MIMAT0018968	Urine	+3.52
MIR3180–1	MIMAT0015058	Urine	+3.39
MIR3663	MIMAT0018085	Urine	+3.14
MIR6824	MIMAT0027548	Urine	+3.01
MIR885	MIMAT0004948	Urine	+2.91
MIR4749	MIMAT0019885	Urine	+2.9
MIR6819	MIMAT0027538	Urine	+2.84
MIR6798	MIMAT0027496	Urine	+2.74
MIR4706	MIMAT0019806	Urine	+2.66
MIR3178	MIMAT0015055	Urine	+2.6
MIR6075	MIMAT0023700	Urine	+2.57
MIR3937	MIMAT0018352	Urine	+2.47
MIR1275	MIMAT0005929	Urine	+2.42
MIR423	MIMAT0004748	Urine	+2.4
MIR6787	MIMAT0027474	Urine	+2.4
MIR6845	MIMAT0027590	Urine	+2.39
MIR4649	MIMAT0019711	Urine	+2.38
MIR6782	MIMAT0027464	Urine	+2.37
MIR3648–1	MI0016048	Urine	+2.36
MIR4674	MIMAT0019756	Urine	+2.32
MIR4758	MI0017399	Urine	+2.31
MIR6808	MIMAT0027516	Urine	+2.3
MIR6090	MI0020367	Urine	+2.3
MIR1909	MIMAT0007883	Urine	+2.29
MIR6848	MIMAT0027596	Urine	+2.29
MIR378H	MIMAT0018984	Urine	+2.27
MIR6802	MIMAT0027504	Urine	+2.24
MIR6750	MIMAT0027400	Urine	+2.23
MIR4466	MI0016817	Urine	+2.23
MIR6510	MIMAT0025476	Urine	+2.21
MIR6778	MIMAT0027456	Urine	+2.2
MIR4750	MIMAT0019887	Urine	+2.16
MIR4673	MIMAT0019755	Urine	+2.15
MIR7150	MIMAT0028211	Urine	+2.14
MIR1224	MIMAT0005458	Urine	+2.13
MIR4467	MIMAT0018994	Urine	+2.09
MIR7110	MIMAT0028117	Urine	+2.09
MIR320E	MI0014234	Urine	+2.08
MIR4417	MIMAT0018929	Urine	+2.05
MIR4274	MIMAT0016906	Urine	+2.02
MIR4492	MIMAT0019027	Urine	+2.02
MIR4701	MIMAT0019799	Urine	–2.08
MIR6812	MIMAT0027524	Urine	–2.12
MIR6124	MIMAT0024597	Urine	–2.13
MIR3613	MIMAT0017990	Urine	–2.15
MIR4440	MIMAT0018958	Urine	–2.16
MIR575	MIMAT0003240	Urine	–2.28
MIR6840	MIMAT0027583	Urine	–2.31
MIR6716	MIMAT0025845	Urine	–2.52
MIR8071–1	MIMAT0030998	Urine	–2.76
MIR320C1	MIMAT0005793	Urine	–2.99
MIR320D1	MIMAT0006764	Urine	–3.19
MIR6790	MIMAT0027480	Urine	–3.58
MIR3188	MIMAT0015070	Urine	–3.63
MIR320A	MIMAT0000510	Urine	–4.2
MIR486–1	MIMAT0002177	Serum	+11.02
MIR149	MIMAT0004609	Serum	+5.57
MIR1237	MIMAT0022946	Serum	+4.94
MIR4687	MIMAT0019775	Serum	+4.84
MIR4463	MIMAT0018987	Serum	+4.38
MIR4529	MIMAT0019068	Serum	+3.93
MIR4508	MIMAT0019045	Serum	+3.28
MIR4763	MIMAT0019913	Serum	+3.15
MIR7706	MI0025242	Serum	+2.82
MIR6756	MIMAT0027412	Serum	+2.8
MIR6776	MI0022621	Serum	+2.72
MIR6715A	MIMAT0025841	Serum	+2.55
MIR6869	MIMAT0027638	Serum	+2.55
MIR3196	MIMAT0015080	Serum	+2.48
MIR409	MIMAT0001639	Serum	+2.43
MIR6750	MIMAT0027400	Serum	+2.37
MIR4728	MIMAT0019849	Serum	+2.13
MIR4700	MIMAT0019797	Serum	+2.09
MIR6819	MIMAT0027539	Serum	+2.06
MIR6803	MIMAT0027506	Serum	– 2.01
MIR4785	MIMAT0019949	Serum	–2.03
MIR320A	MIMAT0000510	Serum	–2.05
MIR4532	MIMAT0019071	Serum	–2.05
MIR4490	MI0016852	Serum	–2.06
MIR1180	MIMAT0026735	Serum	–2.08
MIR3613	MIMAT0017991	Serum	– 2.09
MIR6850	MIMAT0027600	Serum	–2.22
MIR6780B	MIMAT0027572	Serum	–2.28
MIR4668	MIMAT0019745	Serum	–2.45
MIR1908	MIMAT0007881	Serum	–2.48
MIR1228	MIMAT0005582	Serum	–2.52
MIR940	MIMAT0004983	Serum	–2.65
MIR4706	MIMAT0019806	Serum	–2.72
MIR668	MIMAT0026636	Serum	–2.95
MIR602	MIMAT0003270	Serum	–3.00
MIR4497	MIMAT0019032	Serum	–3.19
MIR4707	MIMAT0019807	Serum	–3.96
MIR6511B1	MIMAT0025847	Serum	–5.07
MIR6126	MIMAT0024599	Serum	–18.39
MIR4484	MIMAT0019018	Serum	–23.97

Overlapping miRNAs in all groups of urine and serum samples are summarized in Table 2. Two of the four miRNAs demonstrated significant correlations between the urine and serum samples. In this study, the *MIR4532* (Gene ID:100616353) and *MIR4706* (Gene ID:100616490) were upregulated in urine and downregulated in serum samples. The *MIR320A* (Gene ID:407037) was downregulated in both urine and serum samples (fold change –4.2 and –2.05 respectively); in addition, *MIR6750* (Gene ID:102466192) was upregulated in both urine and serum samples (fold change +2.23 and +2.37, respectively). Moreover, to check the accuracy of the microarray miRNA quantification, the two miRNAs (*MIR320A *and* MIR6750) *were reexamined by using qRT-PCR in 76 samples (38 urine and 38 serum samples from eight patients and 30 healthy controls). The annealing temperatures were evaluated before qRT-PCR reactions and the results were 56 °C for the reference gene (*RNU6-2*), 58 °C for *MIR320A*, and 55 °C for *MIR6750*. 

**Figure 3:**
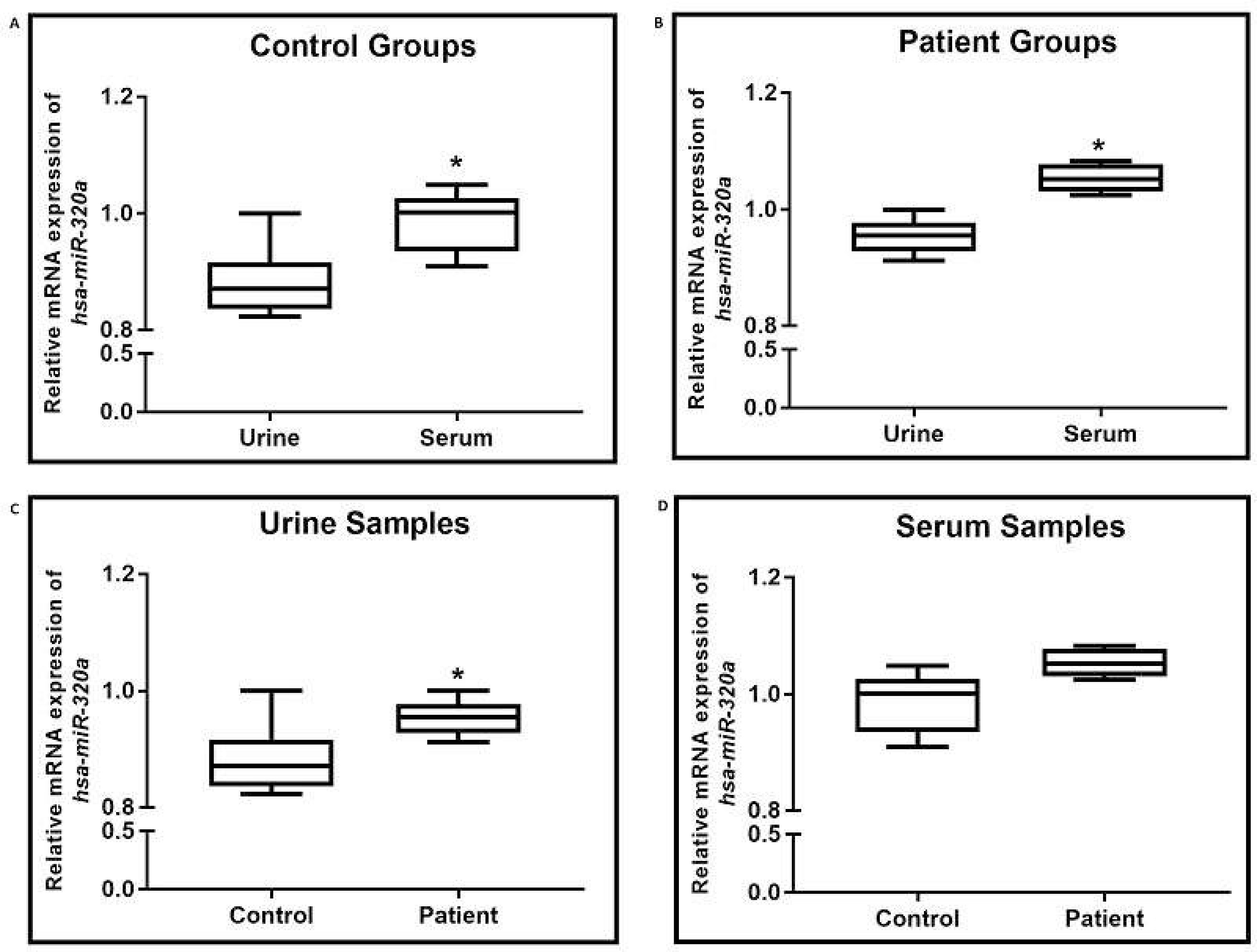
MIR320A expression results. A) Urine and serum sample expression levels of the control group (p = 0.0023). B) Urine and serum sample expression levels of the patient group (p = 0.0026). C) Urine sample expression levels of the control and patient group (p = 0.0168). D) Serum sample expression levels of the control and patient group (p = 0.0826).

The selected candidate miRNAs in urine and serum samples of eight patients were compared. The *MIR320A* expression levels of the urine and serum samples of the healthy controls were higher in serum samples (p = 0.0023), and a similar result was observed in patient samples (p = 0.0026) (Figures 3A and 3B, respectively). In addition, the *MIR320A *expression levels between the patient and healthy control groups were compared, and higher levels were observed in patients (p = 0.0168) (Figure 3C). However, these levels were not found to be significant in serum samples (p > 0.05) (Figure 3D). The *MIR6750* expression levels between urine and serum samples of the healthy controls were not different (p > 0.05) (Figure 4A). The *MIR6750* increased only in serum samples of the patients when compared to urine specimens (p = 0.0079) (Figure 4B). In addition, the comparisons of urine and serum samples between the control and patient groups were not found to be significant (p > 0.05) (Figures 4C and 4D, respectively). 

## 4. Discussion

miRNAs play an important role in the regulation of gene expression through direct binding to the mRNA of protein-encoding genes and affect posttranscriptional suppression of its target genes. Besides, there are several miRNAs which have a role in androgen independence in PC [25,26]. 

Due to the low specificity of current diagnosis criteria, investigations for higher specificity in PC diagnosis still continue while there is a gap for urinary biomarker tests particularly for urologic diseases. Importantly, the observed aberrant miRNA expression in PC patients could be either disease-specific or present in all patients with other urinary diseases [27].

In the present study, we recruited patients with newly diagnosed PC who had not had any treatment yet. Then, we analyzed the entire urinary and serum miRNA expression profiles and compared them with healthy controls. Afterwards, the overlapped and correlated miRNAs were further evaluated by qRT-PCR. The microarray expression data showed that 49 miRNAs were upregulated in urinary samples of PC patients, and 14 miRNAs were downregulated. In addition, up- and downregulated serum samples contain 19 and 21 miRNAs, respectively, when compared with healthy controls as well. The overlapping miRNAs are *MIR4532*, *MIR4706*, *MIR320A*, and *MIR6750* between urine and serum samples which were detected for both patients and healthy controls. However, the microarray results were not found to be completely consistent. According to the overlapping miRNAs between patient and control groups, only two miRNAs (*MIR4532 *and* MIR4706*) were upregulated in urine although they were downregulated in serum samples, which may be due to the different samples being pooled for microarray analysis and/or the possible effects of different ages of the subjects and varying pathological cancer grades of patients. The *MIR4532 *is listed as having possible oncogene regulatory properties which have not been investigated specifically [28]. Only one report demonstrated that *MIR4532 *was found in malignant B cells [29]. However, this miRNA was withdrawn by miRBase, and NCBI reported that the record was discontinued on 14-Mar-2018. On the other hand, *MIR4706* was identified in breast tissue [30], and there were no data for functional properties for this miRNA yet. 

Interestingly, the *MIR320A* was downregulated for both urine and serum samples in microarray expression analysis. On the contrary, urine samples of the patient group showed a significant increase in the expression of *MIR320A *when compared to the control group by qRT-PCR. This result is also consistent with that of a previous study by Porkka et al., which showed upregulation of *MIR320A* in prostate carcinomas when compared to BPH specimens [31]. In addition, Ristou et al. found nine different miRNAs in plasma samples of colorectal cancer patients where *MIR320A* level was upregulated before surgery, and decreased expression was recorded after surgery [32]. Moreover, Okato et al. reported a significant decrease in the expression of *MIR320A *in PC tissue when compared to normal prostate tissue. Furthermore, a previous study proved that cell proliferation, cell migration, and cell invasion are significantly inhibited in the PC cell line by silencing lysosomal-associated protein 1 (LAMP1), which is the target of *MIR320A*; consequently, the data suggested that *MIR320A *may function as a tumor suppressor [33]. It has been recently observed that *MIR320A *suppresses tumor cell proliferation and invasion of renal cancer cells by targeting forkhead box protein M1 (FoxM1) [34]. 

We also analyzed by microarray and verified by qRT-PCR the expression levels of *MIR6750* in serum and urine samples which were not found to be significant between the patient and control group. In one report, *MIR6750 *was listed as human splicing-derived miRNAs (“mirtrons”) [35]. Currently, this is the only data about *MIR6750 *and its relation with PC which was found in both urine and serum samples. However, apart from prostate cancer, only one study in nonsmall cell lung cancer found 59 downregulated miRNAs by silencing the insulin-like growth factor 1 receptor (IGF-1R) gene, and *MIR6750 *has been shown to be downregulated [36]. 

The limitations of our study are that we did not evaluate the cellular sources of miRNAs. Particularly urine specimens have podocytes, inflammatory cells, renal tubular, and urinary tract epithelial cells. In addition, urine samples possibly did not include exosomes which are the major sources of miRNAs in body fluids. Moreover, the low *g* value of centrifugation process also explains the low concentrations of total RNAs from urinary samples. Therefore, the underlying outcome of the changes and correlations observed in this study needs further investigation with more patient samples. 

## 5. Conclusion

There are conflicting results in numerous studies of miRNA expression patterns in prostate cancer. In sum, several studies reported that miRNAs are downregulated in tumors, whereas others reported them to be upregulated [6]. The complexity of miRNAs is not fully clarified either in cancer development or PC in general.

In conclusion, the microarray expression data provided valuable results for possible noninvasive biomarkers for PC patients. Particularly the expression profile of *MIR320A *was significantly altered in urine specimens of the PC patients. Further studies with large cohorts that include both patients and healthy controls are needed to validate the expression profiles of overlapping miRNAs. 

## Abbreviations

BPH: Benign prostate hyperplasia; DRE: Digital rectal examination; FC: Fold change; FDR: False discovery rate; FoxM1: Forkhead box protein M1; HBV: Hepatitis B virus; HCV: Hepatitis C virus; HIV: Human immunodeficiency virus; IGF-1R: Insulin-like growth factor 1 receptor; LAMP1: Lysosomal-associated protein 1; miRNA: Micro RNA; PC: Prostate cancer; PCA: Principal component analysis; PSA: Serum prostate-specific antigen; RMA: Robust multichip average; RT-PCR: Reverse transcription polymerase chain reaction; UTR: Untranslated region

## Informed consent and ethical approval

Informed consent was obtained from all individual participants included in the study. This study was conducted after receiving approval from the Local Human Ethics Committee (approval number: 16926-22/30) from Bezmialem Vakıf University.
